# Total sleep deprivation increases pain sensitivity, impairs conditioned pain modulation and facilitates temporal summation of pain in healthy participants

**DOI:** 10.1371/journal.pone.0225849

**Published:** 2019-12-04

**Authors:** Alexander Torp Staffe, Mathias Winther Bech, Sara Louise Kjær Clemmensen, Henriette Tranberg Nielsen, Dennis Boye Larsen, Kristian Kjær Petersen

**Affiliations:** 1 SMI, Department of Health Science and Technology, Faculty of Medicine, Aalborg University, Aalborg, Denmark; 2 Department of Health Science and Technology, Center for Neuroplasticity and Pain, Faculty of Medicine, Aalborg University, Aalborg, Denmark; University of Modena and Reggio Emilia, ITALY

## Abstract

Chronic pain patients often suffer from insomnia or impaired sleep which has been associated with increased pain sensitivity, but a limited amount of studies have investigated the effects of total sleep deprivation on central pain mechanisms. Therefore, the aim of this study was to determine the effects of total sleep deprivation on temporal summation, conditioned pain modulation, thermal and pressure pain sensitivity in healthy participants. Twenty-four healthy participants took part in this two-session trial. The measurements were conducted after a night of habitual sleep (baseline) and following 24 hours of total sleep deprivation. Detection thresholds for cold and warmth and pain thresholds for cold and heat were assessed. Cuff induced pressure pain detection and tolerance thresholds, temporal summation and conditioned pain modulation were assessed with user-independent, computer-controlled cuff algometry. Conditioned pain modulation was significantly impaired, temporal summation was significantly facilitated and pain sensitivity to pressure and cold pain were significantly increased at follow-up compared with baseline. In conclusion, this study found that one night of total sleep deprivation impaired descending pain pathways, facilitated spinal excitability and sensitized peripheral pathways to cold and pressure pain. Future studies are encouraged to investigate if sleep therapy might normalize pain sensitivity in sleep-deprived chronic pain patients.

## Introduction

The sleep quality declines with various chronic pain conditions as shown in, e.g., fibromyalgia [1], burn injuries [2], and back pain [3]. Studies have reported that at least 50% of patients with diverse chronic pain conditions suffer from sleep impairments [4]. Additionally, sleep impairments have been described as valuable predictors for new incidences and worsening of symptoms linked to chronic pain conditions like fibromyalgia [5], rheumatoid arthritis (RA) [6], and orofacial and back pain [7] as patients with co-occurring sleep impairments seem to experience worsened pain symptoms [4,8].

Though the underlying mechanisms connecting sleep impairments and decreased descending pain inhibitory control are unclear, brain areas such as the periaqueductal gray are known to modulate both sleep stages and nociception [9]. Furthermore, the raphe nuclei are involved in the descending pain control system [10] and the ascending reticular activating system (ARAS), which is responsible for the transition between sleep and wakefulness [11]. To some degree, overlapping functions in these areas of the brain may account for the link between sleep loss and decreased pain inhibition [4,12–15]. However, this encourages further investigation into the connection between sleep loss and central pain processing.

Mechanistic pain profiling assesses the underlying pain mechanisms in the peripheral and central nervous system and includes pain thresholds, temporal summation of pain (TSP), and conditioned pain modulation (CPM) [16]. TSP and CPM are the human surrogate models for wind-up and descending pain inhibitory control, respectively [17,18]. CPM protocols reflect endogenous pain inhibition by measuring the inhibition of a nociceptive stimulus when interceded by a secondary conditioning stimulus (CS), otherwise referred to as the “pain inhibits pain phenomenon”. [17]. An impaired CPM is therefore reflected by a reduced pain inhibits pain effect. It is well-established that patients suffering from back pain [19], fibromyalgia [20], or severe osteoarthritis [21] exhibit reduced descending pain inhibitory control and that impaired CPM is associated with worsening of pain [22]. In addition, CPM has been shown to be impaired following sleep impairment [4,23]. For instance, decreased sleep quality is associated with impaired CPM in healthy participants after three days of fragmented sleep [24] and in patients suffering from temporomandibular joint disorder [25]. TSP mimics the wind-up process, which indicates central sensitization and is interpreted as the gradual increase in pain sensitivity when continuously exposed to stimuli with a constant intensity. Facilitated TSP is reflected by an intensified gradual response to pain stimuli. TSP is facilitated in multiple chronic pain conditions [22] and emerging evidences suggests that facilitated TSP might be a more reliable predictor for poor response to standard pain therapy [16,26–28]. TSP is facilitated in many chronic pain conditions [29–31]. Yet, limited evidence exists on the effect of total sleep deprivation (TSD) on TSP. For example, some studies have observed increased TSP in participants with sleep impairments due to prolonged REM sleep or osteoarthritis [32,33]. In support, one night of TSD increased the thermal and pressure pain sensitivity in healthy participants [34] and 60 hours of sleep deprivation decreased the pressure pain tolerance [35]. Nonetheless, the evidence supporting thermal hyperalgesia is conflicting as some studies finds increased pain sensitivity in both heat pain thresholds (HPT) and cold pain thresholds (CPT) among healthy participants subjected to 24 hours of TSD [36,37]. Contrary to this, other studies report no significant changes in heat pain thresholds among healthy participants after a night of TSD [35,38]. A better understanding of how sleep impairment influences central and peripheral pain mechanisms is important to further develop its use in clinical practice [39], either by implementing sleep therapy or through pharmacological treatment targeting endogenous pain modulation. Therefore, the current study aimed to investigate changes in peripheral pain by applying pressure and assessing the thermal pain thresholds and central pain mechanisms employing TSP and CPM in healthy participants before and after 24 hours of TSD.

## Methods

### Participants

Twenty-five healthy participants (nine women) (average age 22.6 ± 2.02 years) were recruited at Aalborg University through advertisements. The participants received detailed written and oral information and signed an informed consent form prior to enrollment in the experiment. The study was approved by The North Denmark Region Committee on Health Research Ethics (N-20180089) and was conducted in agreement with the Helsinki Declaration.

### Experimental design

The experiment consisted of two identical sessions conducted on two consecutive days. In-between sessions, the participants underwent 24 hours of TSD. To ensure that the participants were awake, they were instructed to send an hourly text message. The experimental pain stimuli were applied in the order of heat, cold, pressure, TSP and CPM. In order to control for order effects, test modalities were conducted in the same order for all participants in both sessions.

### Questionnaires

Prior to the baseline measurements, the participants were asked to report both sleep duration and sleep quality the night before. The participants also filled out the Pittsburgh Sleep Quality index (PSQI) and the Pain Catastrophizing Scale (PCS). The PSQI is a validated tool for assessment of sleep quality [40] in which participants evaluate their sleep based on a four-point Likert-type scale (ranging from 0 = “not at all” to 3 = “three times or more per week”), with high scores indicating poor sleep quality [41]. The PCS questionnaire is a validated tool for assessment of thoughts regarding pain [42,43] based on a five-point Likert-type scale (ranging from 0 = “not at all” to 4 = “all the time”) with a high score indicating a high level of catastrophizing thoughts concerning pain [44].

### Thermal stimuli

Cold and warm detection thresholds (CDT and WDT) and cold and heat pain thresholds (CPT and HPT) were assessed using the Medoc pathway system (Medoc, Israel) in accordance with the protocol of the German Research Network on Neuropathic Pain [45]. Briefly, the 3x3 cm ATS Probe (Medoc, Israel) was placed on the volar forearm, 3 cm below the elbow. Thermal stimuli were applied with ramped stimuli (1°C/s) which were terminated when the participant pressed a button. When assessing CDT or WDT, the participants were instructed to press the stop button as soon as they experienced a change of temperature to either “cool” or “warm”. When assessing CPT or HPT, the participants were instructed to press the stop button immediately following the initial sensation of pain. Cut-off temperatures were 0°C and 50°C and baseline temperature was 32°C. Three consecutive stimuli were applied in each test. The results were averaged to reflect CDT, WDT, CPT, or HPT.

### Pressure stimuli

Cuff pressure detection thresholds (cPDTs), tolerance thresholds (cPTT), TSP, and CPM were assessed by a cuff algometer (Cortex Technology, Hadsund, Denmark, and Aalborg University, Denmark). A cuff was placed on the belly of the gastrocnemius muscle of the participants in agreement with Graven-Nielsen et al. [46]. The cuff was inflated at a progression rate of 1 kPa/s and was set to a maximum limit of 100 kPa. The participants were instructed to rate the level of pain as soon as pain was detected on a visual analogue scale (VAS).

### Pressure pain thresholds

cPDT was defined as the pain-inducing amount of pressure, equivalent to 1 cm on the VAS. The amount of pressure applied causing unbearable pain was defined as cPTT. cPDT and cPTT values were determined as the value of the measurements performed on the dominant leg.

### Temporal summation of pain

TSP was assessed using a mechanical pressure stimulus. The stimuli were applied 10 times with 1 s interstimulus interval and duration [30]. The applied pressure was equal to the cPTT. The participants were instructed to continuously rate their pain on a VAS. An average of the VAS scores measured during the first three pulses and an average of the VAS scores measured during the last three pulses were used to determine TSP. The difference between these averages was interpreted as TSP [47].

### Conditioned pain modulation

On the non-dominant leg, the cuff was promptly inflated to a pressure corresponding to 70% of the cPTT. The cuff on the dominant leg was then inflated at a rate of 1 kPa/s. The participants were instructed to rate the pain on their dominant leg. CPM was calculated as the difference between cPDT with and without the conditioning stimuli. cPDT without the conditioning stimulus was measured as the cuff pressure detection threshold on the dominant leg, when no cuff was inflated on the non-dominant leg.

### Statistics

All statistical analyses were conducted using SPSS version 25 (IBM, USA). For all parameters, including thermal detection thresholds (WDT, CDT), pain sensitivity (HPT, CPT, cPDT, cPTT), and central pain mechanisms (TSP, CPM), separate paired sample t-tests were used to compare the differences of means between measurements (pre-TSD, post-TSD). If the data violated the assumptions for parametric data, a Wilcoxon signed-rank test was performed. Bonferroni correction was applied to account for multiple comparisons when assessing the descending pain inhibitory control (0.05 / 2 comparisons = 0.025). The level of significance was set to *P* ≤ 0.05. Unless otherwise stated, all data are presented as means ± standard deviation (SD).

## Results

### Demographics

Twenty-four healthy participants (eight women) with no history of chronic, mental, musculoskeletal, or neurological illness participated in this study (average age 22.6 ± 2.04 years). Twenty-five participants were initially recruited, but one participant was subsequently excluded due to a previously undisclosed history of mental illness. Due to technical issues during the data collection of CDT, WDT, CPT, and HPT from one participant, the thermal stimuli data are based on 23 participants, whereas TSP, CPM, cPDT, and cPTT are based on 24 participants. Demographics of the 24 participants are presented in **Table 1**.

**Table 1:** An overview of demographics, PSQI, sleep duration, and sleep quality at baseline for all participants (mean ± SD).

Age (years)	22.6 (19–27)
Sex (% females)	33.3%
PSQI	5.04 ± 1.71
Sleep duration before baseline (hours)	6.85 ± 1.11
Habitual sleep duration (hours)	7.25 ± 0.69
Sleep quality before baseline (0–10)	6.92 ± 1.63
Habitual sleep quality (0–10)	7.48 ± 1.44

Data are presented as mean ± SD. PSQI, Pittsburgh Sleep Quality Index. Sleep quality was quantified on a scale from 0 to 10, in which 0 was the worst possible sleep quality and 10 was the best possible sleep quality.

### Thermal stimuli

**Fig 1:**
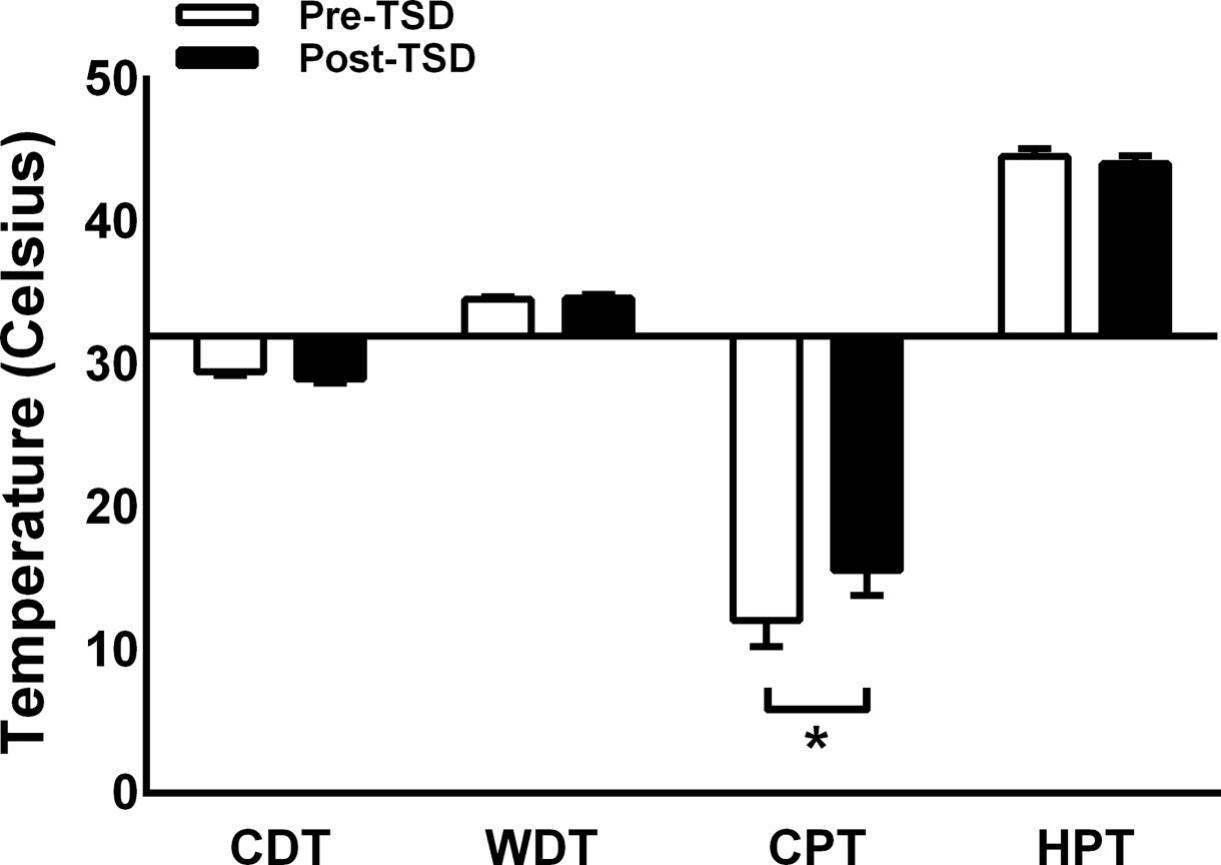
Thermal QST data before and after total sleep deprivation (mean ± SD). A significant decrease in cold pain threshold (CPT) was found after total sleep deprivation (Post-TSD) compared to before TSD (Pre-TSD), whereas cold and warm detection thresholds (CDT and WDT), and heat pain threshold (HPT) were unaffected. *, *p* < 0.05.

No significant changes were found when comparing baseline (CDT: 29.5° Celsius ± 1.03; WDT: 34.6° ± 0.77) and follow-up (CDT: 29° Celsius ± 1.3; WDT: 34.7° ± 1.24) data for CDT (**Fig 1**; *t* = 1.36, *p* = 0.19) and WDT (**Fig 1**; *z* = 0.99, *p* = 0.32). Significantly decreased CPT was observed at follow-up (15.6° ± 8.51) compared with baseline (12.1° ± 8.9, **Fig 1**; *z* = 2.3, *p =* 0.02). Conversely, HPT showed no difference when comparing follow-up (44.1° ± 2.7) with baseline (44.6° ± 2.7, **Fig 1**; *t* = 0.96, *p* = 0.35).

### Pressure pain thresholds

**Fig 2:**
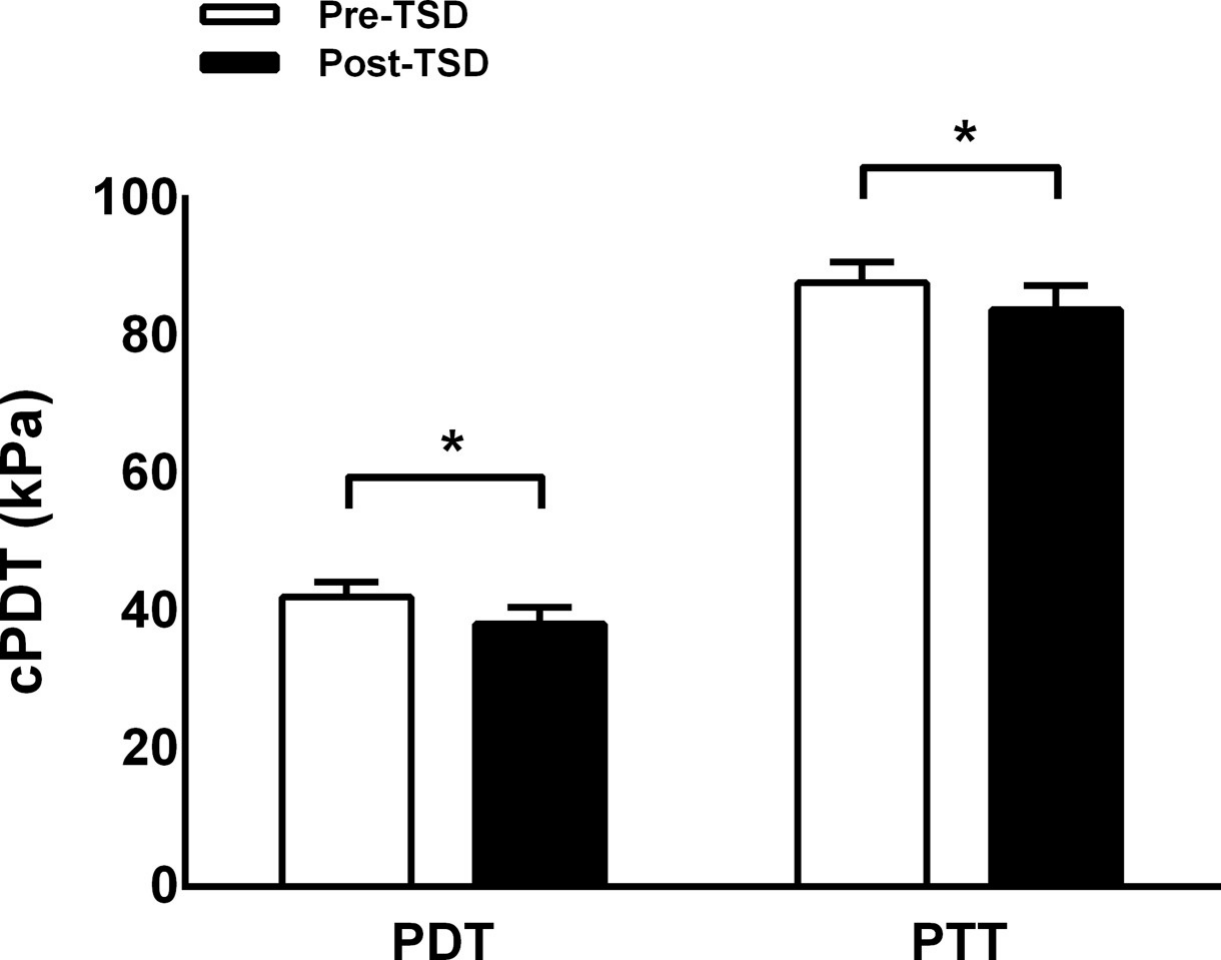
Cuff induced pain detection and tolerance thresholds before and after total sleep deprivation (mean ± SD). Cuff induced pain detection (cPDT) and tolerance thresholds (cPTT) significantly decreased after total sleep deprivation (Post-TSD) compared with before TSD (Pre-TSD). *, *p* < 0.05.

cPDT was significantly reduced after 24 hours of TSD (38.8 kPa ± 11.85) compared with baseline (42.13 kPa ± 10.45, **Fig 2**; *t* = 2.22, *p* = 0.037). Similarly, cPTT was significantly reduced at follow-up (83.9 kPa ± 17.11) compared with baseline (87.83 kPa ± 14.45, **Fig 2**; *z* = -2.11, *p* = 0.03).

### Temporal summation of pain

**Fig 3:**
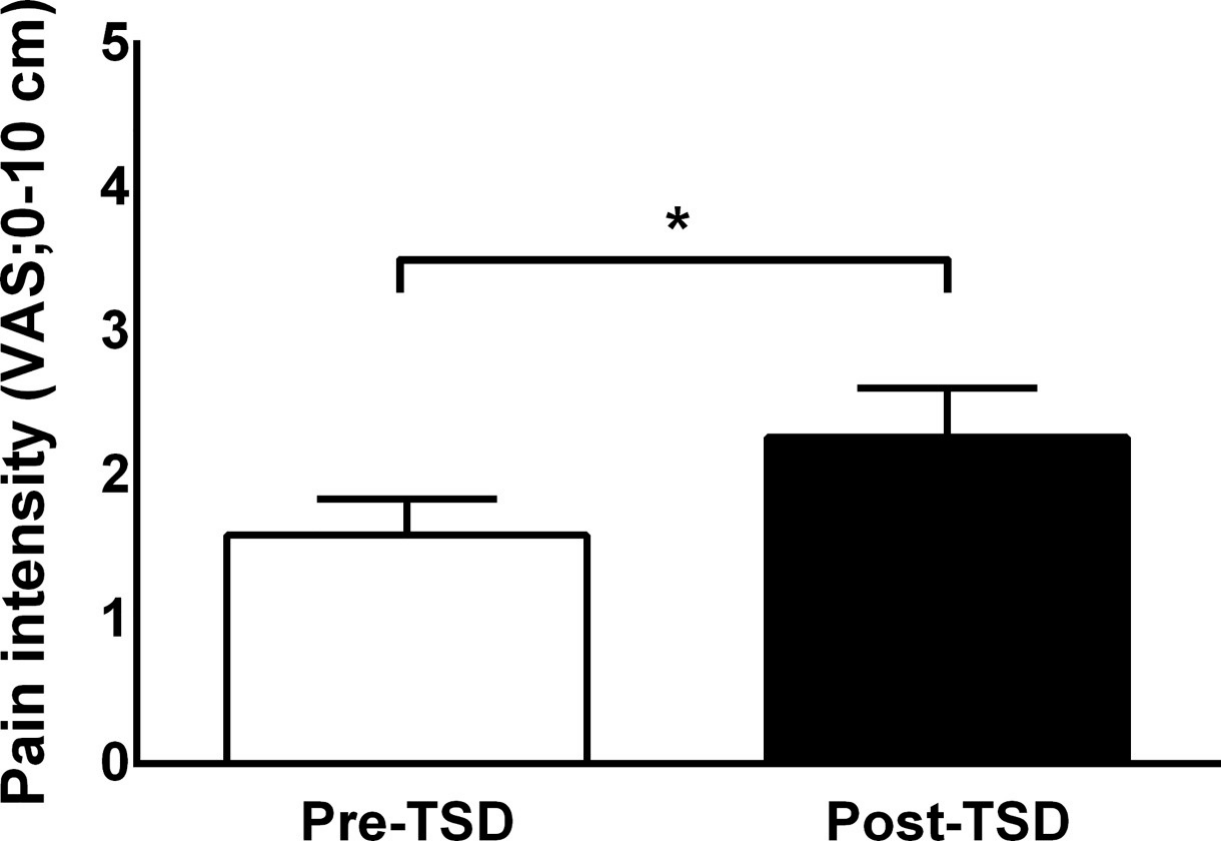
Temporal summation of pain before and after total sleep deprivation (mean ± SD). Temporal summation of pain was significantly increased after total sleep deprivation (Post-TSD) compared to before TSD (Pre-TSD). *, *p* < 0.05.

TSP was significantly facilitated at follow-up (2.27 VAS ± 1.66) compared with baseline (1.59 VAS ± 1.23, **Fig 3**; *t* = -2.68, *p* = 0.01).

### Conditioned pain modulation

**Fig 4:**
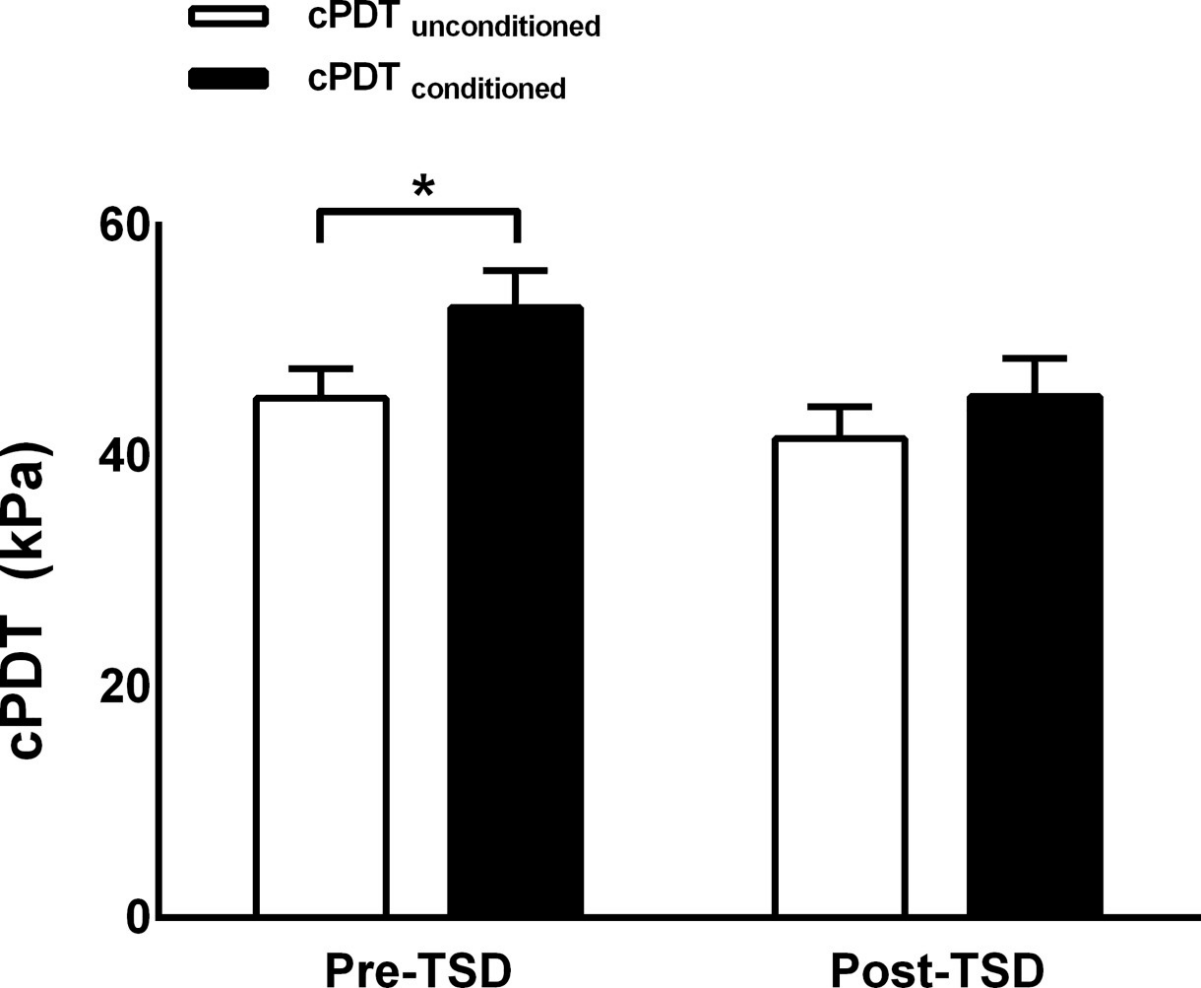
Conditioned pain modulation before and after total sleep deprivation (mean ± SD). Conditioned pain modulation is assessed using an unconditioned (white bars) and conditioned cuff pain detection threshold (cPDT). Subjects displayed a significant (*, P<0.05) increase in conditioned cPDT compared with unconditioned cPDT at baseline but not following 24 hours of total sleep deprivation (TSD).

cPDT was significantly increased with conditioning stimulus compared with cPDT without conditioning stimulus at baseline (**Fig 4**; *t* = -3.63, *p* = 0.002, Bonferroni-corrected). Conversely, cPDT did not increase during conditioning after 24 hours of TSD (*t* = -1.81, *p* = 0.168, Bonferroni-corrected).

## Discussion

This study is the first to demonstrate that 24-hours of TSD impairs CPM, facilitates TSP, and increases pain sensitivity to pressure and cold pain stimuli in one experimental setup, indicating that TSD affects both central and peripheral pain pathways.

### Sleep impairment and descending pain inhibitory control

An association between impaired CPM and decreased sleep quality has been shown in several studies investigating sleep impairment in patients with conditions such as RA [6], fibromyalgia [48], and insomnia [49]. The current study sought to investigate whether a similar relationship was present in healthy participants as it would indicate whether the relationship between sleep and pain modulation is limited to chronic pain conditions.

The current study found impaired CPM after 24 hours of TSD, which is in line with a recent study conducted by Eichhorn et al. [50], who demonstrated impaired descending pain inhibitory control following one night of TSD. In contrast, Smith et al. [51] assessed CPM and found reduced descending pain inhibition after partial sleep deprivation (PSD) but found no alterations in CPM following 36 hours of TSD. Finally, Matre et al. [52] found a significantly improved CPM following PSD. These conflicting results concerning the effect of sleep deprivation on CPM may be explained by the heterogeneity in the methodological approaches to the CPM protocols [53]; the current study used cuff algometry whereas earlier studies used a cold-pressor test.

The findings of the current study support the theory that sleep impairments decrease the effect of the descending pain inhibition. The underlying mechanism of the interaction between sleep impairments and impaired descending pain inhibition remains unclear. However, a theory may be that brain areas such as the periaqueductal gray and the raphe nuclei both are involved in the descending pain control system [10], nociception [9], and modulation of sleep [11]. Therefore, neurotransmitter alterations in these areas due to sleep impairments could cause a decrease in the effect of descending pain inhibitory control. This could explain why sleep impairments are often seen in chronic pain patients.

A better understanding of the interaction between sleep and central pain mechanisms could possibly improve the treatment options for chronic pain patients since sleep impairment is a major problem among chronic pain patients [6,48,49]. Future studies are encouraged to investigate whether the effects of sleep impairments on central pain mechanisms are reversible through sleep therapy.

### Sleep impairment and temporal summation of pain

Central sensitization, demonstrated as TSP, represents an essential pathophysiological process that augments the development and maintenance of pain conditions in several clinical contexts [30,54]. TSP has been found to be increased in women suffering from insomnia [32] and in individuals with increased REM-sleep [33]. In contrast, Schuh-Hofer et al. [34] found increased sensitivity to pinprick stimuli and hyperalgesia to cold but found no changes in TSP. Similarly, no association was found between increased TSP and sleep impairments in patients with primary insomnia [49], fibromyalgia [55], and restless legs syndrome [56]. However, previous studies tested TSP using heat pain, and since pressure pain is primarily mediated by A-fiber nociceptor inputs [57] while heat pain depends mainly on C-fiber inputs [58], it was hypothesized that targeting different nociceptive pathways might produce separate results. The current study found that pressure-induced TSP was facilitated following 24 hours of TSP, which is in contrast to previous findings. This could be explained by different methodological setups and by the fact that the aforementioned studies included patients as subjects. Facilitated TSP following 24 hours of TSD indicates increased central sensitization of pain following sleep loss. Sleep therapy may be a possible invention method for pain relief.

### Sleep and pain sensitivity

A meta-analysis by Schrimpf et al. [59] demonstrated that decreased sleep quality was associated with increased pain sensitivity across a range of different test modalities such as pressure algometry, laser stimuli, and thermal tests. In this respect, studies have reported that CPT increased [36] and HPT decreased [60] following TSD. Additionally, decreased sleep quality has also been linked to decreased mechanical pain thresholds [34,61,62]. For instance, Chiu et al. [61] found a decrease in PPTs in participants with self-reported poor sleep, while Aǧargün et al. [63] found a negative correlation between pain and sleep quality as poor sleep led to an increase in pain sensitivity in patients with fibromyalgia.

Increased pain sensitivity has also been reported following experimentally induced sleep deprivation as Azevedo et al. [64] found a significant increase in thermal and mechanical sensitivity after two nights of TSD. Schuh-Hofer et al. [34] found that only one night of TSD was able to promote a state of generalized hyperalgesia among the participants. Like the current study, Schuh-Hofer et al. found no alterations in CDT and WDT. The fact that no changes were demonstrated in non-nociceptive stimulations led Schuh-Hofer et al. [34] to conclude that the sensory alterations following sleep loss were purely nociceptive. The hyperalgesic effects of 24 hours of TSD in healthy participants have also been demonstrated using a cold pressor test [65,66] and radiant heat laser pulses [64]. Together with the results of this study, these previous results support the notion that TSD causes generalized hyperalgesia detectable across a number of QST modalities.

Even reduced sleep duration seems to affect the pain sensitivity as participants with a short sleep duration (< 6 hours) reported increased pain sensitivity the day following the short sleep duration [67]. These results indicate that while TSD does increase the pain sensitivity, even disturbed sleeping patterns may cause the pain sensitivity to increase. This sentiment is supported by Onen et al. [35], who tested the pain sensitivity following TSD as well as PSD. While a significantly decreased cPDT was found after 40 hours of PSD, Onen et al. [35] found the hypersensitivity to be more extensive after TSD compared with PSD, supporting that both sleep loss and impairments increase the pain sensitivity. The connection between sleep restriction and pain sensitivity has also been applied to clinical trials as Fitzgerald et al. [68] found increased pain sensitivity when examining self-reported pain in RA patients after PSD. Though multiple sleep disturbance paradigms have produced results in the past, the current study applied TSD as it seems to produce hyperalgesia more extensively and consistently than PSD.

In contrast, Arima et al. [69] reported no interaction between partial sleep deprivation and pain sensitivity and Onen et al. [35] found no significant changes in thermal pain after TSD and PSD. However, Onen et al. [35] found hyperalgesia to mechanical stimuli and suggested that these results might have been affected by undetected differences in skin surface temperatures or the fact that thermal tests generally show less reliably when it comes to discriminating small changes in pain thresholds [70].

A generalized hyperalgesic effect from TSD is widely reported [34], with mechanical stimulus tests seemingly producing the most consistent results. The findings presented in the current study support earlier results as the participants exhibited mechanic and thermal hyperalgesia following 24 hours of TSD.

### Limitations

This study monitored TSD by hourly text messages and this procedure does not guarantee that the participants did not sleep in between. However, one study observed that partial sleep deprivation has a greater effect than TSD regarding CPM [51]. Therefore, it is unlikely to have impacted the current findings even if some participants did fall asleep. The participants in the current study scored an average of more than 5 on the PSQI and averaged 6.85 hours of sleep the night before the baseline session. The fact that the sample in this study displayed both lowered general sleep quality and average sleep duration the night before baseline suggests that this current sample may be less sensitive to sleep deprivation. Despite this, it is noteworthy that the current study still demonstrated an effect on the central pain mechanisms. A sample with higher average sleep quality and sleep duration closer to recommended amounts may have produced an even more profound hyperalgesic response.

## Conclusion

The current study is the first to demonstrate that TSD impairs CPM, facilitates TSP, and increases pain sensitivity to pressure and cold pain stimuli, indicating that TSD affects both central and peripheral pain pathways. Future studies are encouraged to investigate the underlying mechanism of TSP, the effects on both the central and peripheral nervous system, and whether the effects are reversible through sleep therapy.

## Supporting information

S1 Table cPDT and cPTT. Raw data for cuff pressure pain detection & tolerance thresholds before and after total sleep deprivation.
(DOCX)

S2 Table CPM. Raw pressure detection thresholds (conditioned and unconditioned) measured before and after total sleep deprivation.
(DOCX)

S3 Table TSP. Difference between the first and last three pain intensity ratings during temporal summation of pain.
(DOCX)

S4 Table Thermal QST. Averaged cold detection threshold, warm detection threshold, cold pain threshold, and heat pain threshold values before and after total sleep deprivation.
(DOCX)
