# Supplementary material for: Total sleep deprivation increases pain sensitivity, impairs conditioned pain modulation and facilitates temporal summation of pain in healthy participants
Source: PLoS One. 2019 Dec 4;14(12):e0225849. doi: 10.1371/journal.pone.0225849 (PMC6892491; doi:10.1371/journal.pone.0225849)
Supplement: S1 Table — Raw data for cuff pressure pain detection & tolerance thresholds before and after total sleep deprivation. (DOCX) [file pone.0225849.s001.docx]

**S1. cPDT and cPTT**

|  | **cPDT** | | **cPTT** | |
| --- | --- | --- | --- | --- |
|  | **Pretest** | **Posttest** | **Pretest** | **Posttest** |
| **1** | 41.85 | 25.4 | 86.65 | 75.95 |
| **2** | 47.65 | 42.4 | 66.75 | 57.7 |
| **3** | 43.85 | 54.3 | 100.35 | 100.25 |
| **4** | 39.8 | 45.7 | 100.4 | 100.3 |
| **5** | 36.85 | 16.85 | 100.05 | 93.35 |
| **6** | 41.85 | 37.25 | 100.3 | 100.05 |
| **7** | 41.25 | 32.45 | 94.65 | 86.45 |
| **8** | 56.7 | 63.9 | 100.3 | 100.25 |
| **9** | 53.95 | 38.9 | 100.2 | 100.1 |
| **10** | 28.6 | 36.15 | 94.4 | 100.15 |
| **11** | 48.6 | 41.25 | 100.15 | 100.3 |
| **12** | 13.35 | 23.05 | 57.6 | 65.5 |
| **13** | 54 | 45.65 | 91.4 | 94.1 |
| **14** | 40.05 | 26.95 | 90.6 | 60.9 |
| **15** | 52.45 | 43 | 95 | 86.3 |
| **16** | 45.3 | 35 | 97.65 | 79.55 |
| **17** | 43.6 | 43.4 | 86.5 | 89 |
| **18** | 37.25 | 30.7 | 84.6 | 82 |
| **19** | 35.95 | 40.85 | 66.05 | 67.75 |
| **20** | 61.55 | 59.45 | 100.25 | 99.95 |
| **22** | 41.5 | 45.9 | 77.5 | 85.5 |
| **23** | 32.45 | 21.3 | 59.7 | 42.85 |
| **24** | 30.6 | 28.45 | 68.95 | 61.45 |
| **25** | 42.1 | 38.7 | 95.5 | 87.1 |
|  |  |  |  |  |
| Mean | 42.12917 | 38.20625 | 88.14583 | 84.03333 |
| SD | 10.22145 | 11.58741 | 14.21749 | 16.74767 |
| SEM | 2.086445 | 2.365271 | 2.902134 | 3.418604 |
