# Supplementary material for: Total sleep deprivation increases pain sensitivity, impairs conditioned pain modulation and facilitates temporal summation of pain in healthy participants
Source: PLoS One. 2019 Dec 4;14(12):e0225849. doi: 10.1371/journal.pone.0225849 (PMC6892491; doi:10.1371/journal.pone.0225849)
Supplement: S2 Table — Raw pressure detection thresholds (conditioned and unconditioned) measured before and after total sleep deprivation. (DOCX) [file pone.0225849.s002.docx]

**S2. CPM**

| **Before TSD** | |  |  | **After TSD** |  |  |  |
| --- | --- | --- | --- | --- | --- | --- | --- |
|  | **PDT** | **Conditioned PDT** | |  | **PDT** | **Conditioned PDT** | |
| **1** | 49 | 35.8 |  | **1** | 26.1 | 26.2 |  |
| **2** | 41.6 | 59.6 |  | **2** | 47.2 | 57.2 |  |
| **3** | 51 | 76.8 |  | **3** | 64 | 83.5 |  |
| **4** | 41.7 | 67.7 |  | **4** | 50.3 | 56.9 |  |
| **5** | 44.1 | 35.5 |  | **5** | 16.4 | 8.3 |  |
| **6** | 38.3 | 36.6 |  | **6** | 37.3 | 54.8 |  |
| **7** | 39.3 | 45.2 |  | **7** | 32 | 15.8 |  |
| **8** | 62 | 69.9 |  | **8** | 74.1 | 76.2 |  |
| **9** | 60 | 73.6 |  | **9** | 42.3 | 39 |  |
| **10** | 24.9 | 29.8 |  | **10** | 36 | 44.6 |  |
| **11** | 62.1 | 71 |  | **11** | 49.3 | 54.1 |  |
| **12** | 14.7 | 32 |  | **12** | 31.9 | 32.8 |  |
| **13** | 52.9 | 72.8 |  | **13** | 47.4 | 50.7 |  |
| **14** | 40.2 | 53 |  | **14** | 32.1 | 44.2 |  |
| **15** | 54.9 | 57.2 |  | **15** | 42.7 | 40.3 |  |
| **16** | 46 | 55.7 |  | **16** | 39 | 45.9 |  |
| **17** | 50.1 | 52.9 |  | **17** | 37.6 | 54 |  |
| **18** | 45.4 | 58 |  | **18** | 38 | 39.4 |  |
| **19** | 36.9 | 48.9 |  | **19** | 49.9 | 47.9 |  |
| **20** | 72.4 | 78.7 |  | **20** | 67.4 | 51.4 |  |
| **22** | 37.8 | 38.8 |  | **22** | 45.7 | 39.2 |  |
| **23** | 33.1 | 42.7 |  | **23** | 22.1 | 43.9 |  |
| **24** | 34.9 | 44.4 |  | **24** | 30.4 | 33.3 |  |
| **25** | 47.5 | 32 |  | **25** | 37.1 | 44.7 |  |
|  |  |  |  |  |  |  |  |
|  |  |  |  |  |  |  |  |
| Mean | 45.03333 | 52.85833 |  |  | 41.5125 | 45.17917 |  |
| SD | 12.52794 | 15.75999 |  |  | 13.63204 | 16.221 |  |
| SEM | 2.557255 | 3.216995 |  |  | 2.782628 | 3.311098 |  |
