# Supplementary material for: Total sleep deprivation increases pain sensitivity, impairs conditioned pain modulation and facilitates temporal summation of pain in healthy participants
Source: PLoS One. 2019 Dec 4;14(12):e0225849. doi: 10.1371/journal.pone.0225849 (PMC6892491; doi:10.1371/journal.pone.0225849)
Supplement: S3 Table — Difference between the first and last three pain intensity ratings during temporal summation of pain. (DOCX) [file pone.0225849.s003.docx]

**S3. TSP**

|  | **TSP before TSD** | **TSP after TSD** |
| --- | --- | --- |
| **1** | 5.266667 | 5.5 |
| **2** | 1.733333 | 3.933333 |
| **3** | 2.4 | 1.766667 |
| **4** | 0.566667 | 1.2 |
| **5** | 1.333333 | 0.433333 |
| **6** | -0.16667 | 1.966667 |
| **7** | 0.9 | 4.966667 |
| **8** | 0.3 | 0.933333 |
| **9** | 2.2 | 1.466667 |
| **10** | -0.06667 | 1.266667 |
| **11** | 1.4 | 3.1 |
| **12** | 1.966667 | 1 |
| **13** | 1.1 | 1.433333 |
| **14** | 2.766667 | 4.933333 |
| **15** | 1.133333 | 2.466667 |
| **16** | 2.566667 | 3.633333 |
| **17** | 0.733333 | 0.533333 |
| **18** | 1.666667 | 1.5 |
| **19** | 0.133333 | 0.4 |
| **20** | 0.433333 | -0.06667 |
| **22** | 3.133333 | 4.8 |
| **23** | 1.733333 | 1.2 |
| **24** | 2.766667 | 3.8 |
| **25** | 2.133333 | 2.233333 |
|  |  |  |
| Mean | 1.588889 | 2.266667 |
| SD | 1.231772 | 1.657919 |
| SEM | 0.251434 | 0.338421 |
