# Supplementary material for: Total sleep deprivation increases pain sensitivity, impairs conditioned pain modulation and facilitates temporal summation of pain in healthy participants
Source: PLoS One. 2019 Dec 4;14(12):e0225849. doi: 10.1371/journal.pone.0225849 (PMC6892491; doi:10.1371/journal.pone.0225849)
Supplement: S4 Table — Averaged cold detection threshold, warm detection threshold, cold pain threshold, and heat pain threshold values before and after total sleep deprivation. (DOCX) [file pone.0225849.s004.docx]

**S4. Thermal QST**

|  | **CDT before TSD** | **CDT after TSD** | **WDT before TSD** | **WDT after TSD** | **CPT before TSD** | **CPT after TSD** | **HPT before TSD** | **HPT after TSD** |
| --- | --- | --- | --- | --- | --- | --- | --- | --- |
| **1** | 30.4 | 28 | 34.1 | 35.7 | 14.6 | 19.3 | 44.6 | 45.1 |
| **2** | 29.4 | 28.7 | 34.9 | 34.8 | 0 | 0 | 48.7 | 47.4 |
| **3** | 29.5 | 26 | 35.2 | 36.3 | 27.4 | 23.4 | 42.1 | 41.2 |
| **4** | 29.9 | 28.5 | 34.3 | 34.9 | 8.6 | 9.9 | 39.2 | 43.7 |
| **5** | 29.3 | 30.4 | 34.1 | 34.5 | 25 | 26.9 | 45.5 | 44.7 |
| **6** | 28.8 | 28.4 | 34.7 | 36.2 | 6.9 | 22.9 | 47 | 41.8 |
| **7** | 29 | 27.7 | 34.2 | 35.7 | 5.4 | 19 | 47.1 | 45.3 |
| **8** | 30.8 | 29.5 | 34.3 | 34.6 | 0.5 | 5.9 | 46.6 | 45.2 |
| **9** | 29.8 | 29 | 34.1 | 35.4 | 8.4 | 13.6 | 45.2 | 44.6 |
| **10** | 30.1 | 28 | 33.9 | 36.1 | 13 | 8.3 | 46 | 46 |
| **11** | 27.5 | 29.3 | 34.3 | 34.1 | 0 | 0.7 | 47.9 | 46.9 |
| **12** | 30.1 | 30.6 | 34.6 | 34.6 | 0 | 0 | 46.7 | 45.7 |
| **13** | 30.4 | 26.9 | 34.4 | 35 | 16.3 | 16 | 39.8 | 46.6 |
| **14** | 29.8 | 30.5 | 34.7 | 34.8 | 2.1 | 21.5 | 42.4 | 41.1 |
| **15** | 30.2 | 31.4 | 35.7 | 33.8 | 21.1 | 18.2 | 46.1 | 46 |
| **16** | 27.8 | 29.6 | 34.4 | 35.8 | 24.3 | 26.2 | 41.3 | 39.4 |
| **18** | 26.9 | 28.9 | 34.3 | 30.4 | 22.1 | 23.9 | 41.3 | 40.7 |
| **19** | 29.1 | 28.4 | 35.1 | 33.9 | 14.6 | 19.4 | 44.5 | 46.8 |
| **20** | 29.9 | 30.8 | 34.1 | 34.1 | 14.5 | 23.8 | 44.4 | 37.8 |
| **22** | 28.5 | 28 | 34.1 | 34.5 | 9.3 | 6.5 | 46.6 | 47 |
| **23** | 31.2 | 29.5 | 33.6 | 33.7 | 22.4 | 20.2 | 47.5 | 45.4 |
| **24** | 29.6 | 30.3 | 35 | 33.8 | 17.2 | 20 | 44.7 | 44.2 |
| **25** | 29.4 | 28.6 | 37.4 | 35.3 | 4.4 | 13.5 | 40.8 | 41.1 |
|  |  |  |  |  |  |  |  |  |
| Mean | 29.45217 | 29 | 34.58696 | 34.69565 | 12.0913 | 15.61304 | 44.6087 | 44.07391 |
| SD | 1.033032 | 1.309754 | 0.776533 | 1.234901 | 8.888037 | 8.506858 | 2.742579 | 2.710286 |
| SEM | 0.215402 | 0.273103 | 0.161918 | 0.257495 | 1.853284 | 1.773803 | 0.571867 | 0.565134 |
